# Supplementary material for: As(III, V) Uptake from Nanostructured Iron Oxides and Oxyhydroxides: The Complex Interplay between Sorbent Surface Chemistry and Arsenic Equilibria
Source: Nanomaterials (Basel). 2022 Jan 20;12(3):326. doi: 10.3390/nano12030326 (PMC8840107; doi:10.3390/nano12030326)
Supplement: Supplementary file 1 [file nanomaterials-12-00326-s001.zip › SI_nanomaterials-1537170.pdf]

nanomaterials

MDPI

Supplementary Material

As(III, V) Uptake from Nanostructured Iron Oxides and Oxyhydroxides: The Complex Interplay between Sorbent Surface Chemistry and Arsenic Equilibria

Marco Sanna Angotzi [1,2], Valentina Mameli [1,2,*], Alessandra Fantasia [1], Claudio Cara [1,2], Fausto Secci [1,2], Stefano Enzo [3], Marianna Gerina [4] and Carla Cannas [1,2]

[1] Department of Chemical and Geological Sciences, University of Cagliari, S.S. 554 bivio per Sestu, 09042 Monserrato, Italy; marcosanna@unica.it (M.S.A.); fantasia.91@hotmai.it (A.F.); cara.claudio16@gmail.com (C.C.); fausto.s1993@gmail.com (F.S.); ccannas@unica.it (C.C.)

[2] Consorzio Interuniversitario Nazionale per la Scienza e Tecnologia dei Materiali (INSTM), Via Giuseppe Giusti 9, 50121 Firenze, Italy

[3] Department of Chemistry and Pharmacy, University of Sassari, Via Vienna 2, 07100 Sassari, Italy; enzo@uniss.it

[4] Department of Inorganic Chemistry, Charles University, Hlavova 8, 12800 Prague 2, Czech Republic; marygerina@hotmail.it

* Correspondence: valentina.mameli@unica.it

Citation: Sanna Angotzi, M.; Mameli, V.; Fantasia, A.; Cara, C.; Secci, F.; Enzo, S.; Gerina, M.; Cannas, C. As(III, V) Uptake from Nanostructured Iron Oxides and Oxyhydroxides: The Complex Interplay between Sorbent Surface Chemistry and Arsenic Equilibria. *Nanomaterials* **2022**, *12*, x. https://doi.org/10.3390/xxxxx

Academic Editor(s): Zheng Ling

Received: 17 December 2021
Accepted: 18 January 2022
Published: date

Publisher's Note: MDPI stays neutral with regard to jurisdictional claims in published maps and institutional affiliations.

Copyright: © 2022 by the authors. Submitted for possible open access publication under the terms and conditions of the Creative Commons Attribution (CC BY) license (https://creativecommons.org/licenses/by/4.0/).

Table of Contents

1. Experimental Details 2
2. Characterization of the sorbents 2
3. Theoretical behaviour of the sorbents and the arsenic species 6
4. Adsorption tests: effect of pH As^{V} 8
5. Adsorption tests: effect of pH As^{III} 13
6. FTIR Spectra 17
7. Adsorption tests: effect of initial concentration 18
8. Adsorption tests: effect of contact time 19
9. Adsorption tests: effect of ionic strength 21
10. Adsorption tests: effect of competitors 22

1. Experimental Details

Table S1. Experimental parameters for adsorption tests. The sorbent amount was 50 mg and solution volume 20 mL for all the tests.

Sorb.	Effect	C_0 As^V (mg L^{-1})	C_0 As^{III} (mg L^{-1})	t (min)	pH_0	NaCl (M)	As/Comp (mol/mol)
**Aka, Fer, Mag, Comp**	pH	100	0	960	2, 3, 4, 6, 8	0	0
**Aka, Fer, Mag, Comp**	pH	0	100	960	2, 3, 4, 6, 8	0	0
**Aka, Fer**	pH	500	0	960	2, 3, 4, 6, 8	0	0
**Aka, Fer**	pH	0	500	960	2	0	0
**Aka**	C_0	10, 50, 100, 150, 200, 250, 350, 500	0	960	3, 8	0	0
**Aka**	Time	250	0	10, 30, 60, 120, 240, 960	3, 8	0	0
**Aka**	Ionic Strength	250	0	960	3, 8	0, 0.01, 0.1, 1	0
**Aka**	Competitor	250	0	960	3, 8	0	1:1, 1:10, 1:100

Sorbent name, studied effet, initial concentration (C_0), contact time, initial pH (pH_0), ionic strength concentration (NaCl), arsenic/competitor ratio (SO_4^{2-}, PO_4^{3-}).

2. Characterization of the sorbents

Table S2. Hyperfine parameters obtained by fitting procedure of the ^{57}Fe Mössbauer spectra of the sorbents.

Sample	Subspectrum	IS (mm s^{-1})	QS (mm s^{-1})	B_{Hf} (T)	W (mm s^{-1})	D	A (%)
**Aka**	Doublet	0.373(1)	0.536(7)	-	0.330(7)	0.095(4)	57
	Doublet	0.379(2)	0.940(9)	-	0.346(8)	0.071(4)	43
**Fer (1st attempt)**	Doublet	0.338(2)	0.697(4)	-	0.615(6)	0.1977(9)	-
**Fer (2nd attempt)**	Doublet	0.321(8)	0.38(3)	-	0.27(7)	0.02(1)	10
	Doublet	0.340(3)	0.74(4)	-	0.59(1)	0.17(1)	88
	Doublet	0.34(1)	1.3(5)	-	0.2(1)	0.005(4)	2
**Mag**	Sextet	0.326(2)	0.007(6)	47.09(3)	0.44(1)	0.13(1)	50
	Sextet	0.328(3)	-0.043(8)	41.9(4)	0.58(2)	0.13(1)	50
**Comp**	Doublet	0.34(1)	0.74(2)	-	0.61(2)	0.008(1)	-

IS is isomer shift. QS is the quadrupole splitting, B_{HF} is the hyperfine field; W is the width; D is the depth; A is the area.

Table S3. FTIR bands of the sorbents.

Aka		Mag		Fer		Comp	
Wavenumber (cm^{-1})	Vibration mode	Wavenumber (cm^{-1})	Vibration mode	Wavenumber (cm^{-1})	Vibration mode	Wavenumber (cm^{-1})	Vibration mode
**3300**	ν H_2O	**3300**	ν H_2O	**3400**	ν H_2O	**3400**	ν H_2O
**1615**	δ H_2O	**1620**	δ H_2O	**1630**	δ H_2O	**1630**	δ H_2O
**1360**	ν Fe-OH	**820**	ν Fe-O	**1500**	ν Fe-OH	**1220**	ν Si-O-Si
**1100**	ν Fe-OH	**630**	ν Fe-O	**1330**	ν Fe-OH	**1090**	ν Si-O-Si
**850**	λ OH-Cl	**570**	ν Fe-O	**1065**	ν Fe-OH	**970**	ν Si-O-H
**680**	ν_{as} Fe-O			**850**	ν Fe-OH	**810**	ν Si-O-Si
**470**	ν_s Fe-O			**600**	ν Fe-O	**470**	ν Fe-O
				**440**	ν Fe-O		

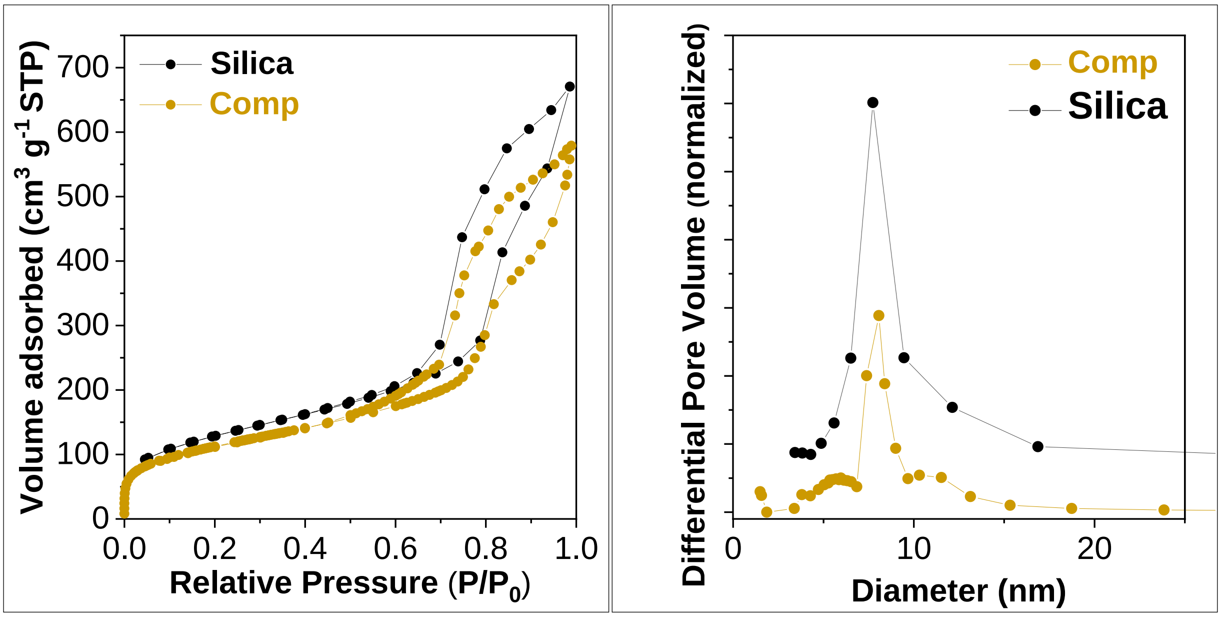

Figure S1. N_2-physisorption isotherms (left) and BJH-calculated pore size distributions (right) of the silica-based samples.

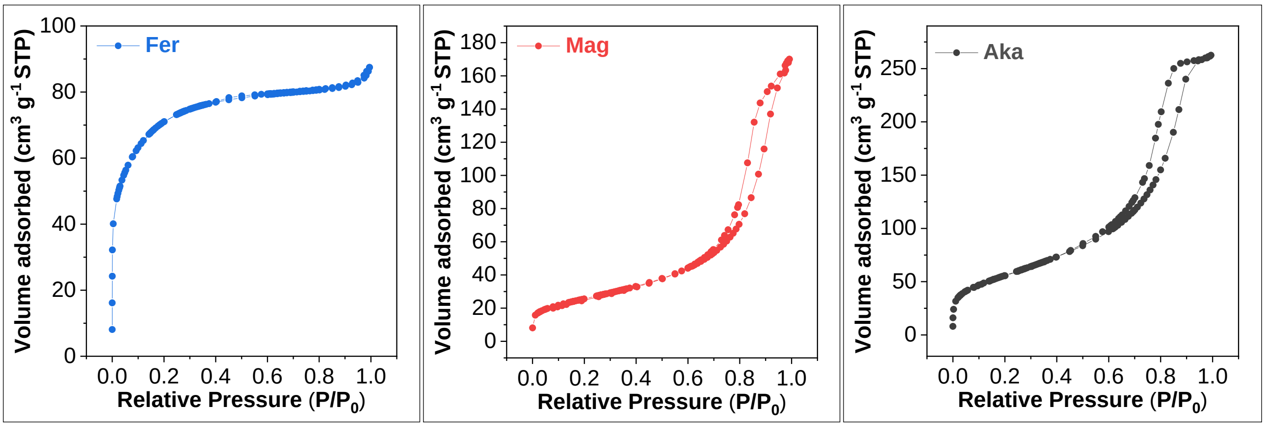

Figure S2. N_2-physisorption isotherms of the iron oxide sorbents.

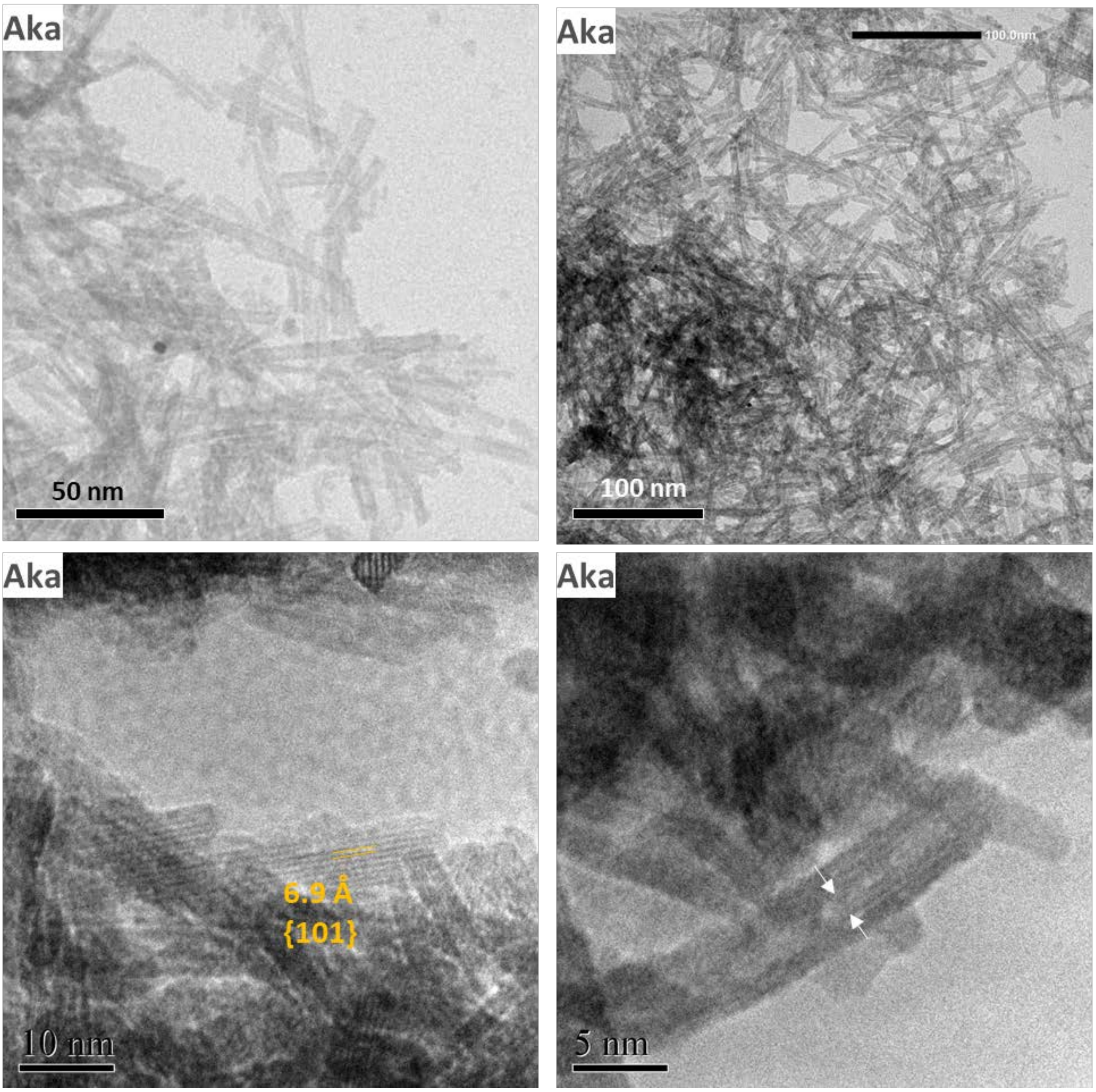

Figure S3. TEM and HRTEM micrographs of Aka nanorods.

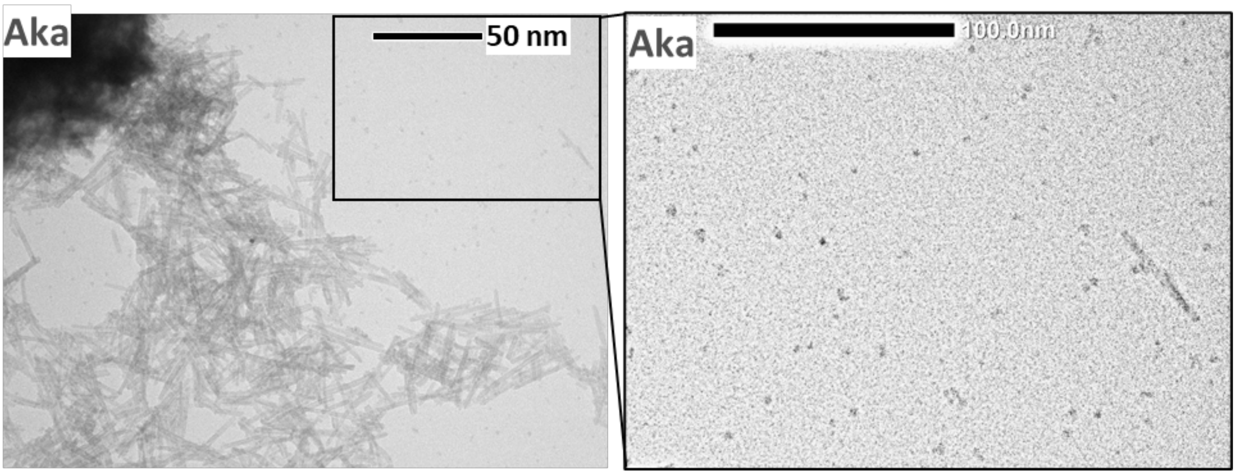

Figure S4. TEM micrograph of Aka and magnification to highlight the small nanoparticles.

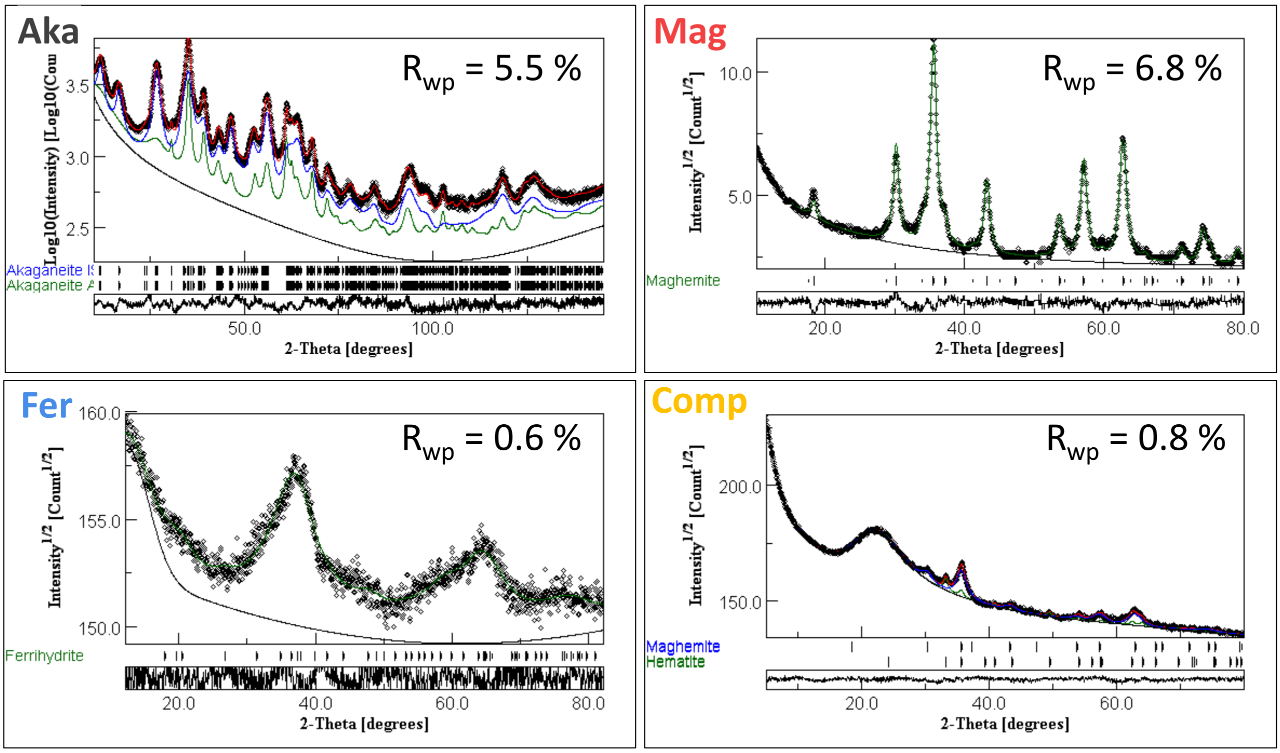

Figure S5. Rietveld refinement of XRD patterns of the samples. Inset of crystal shape modelled from Popa rule for the Aka sample.

3. Theoretical behaviour of the sorbents and the arsenic species

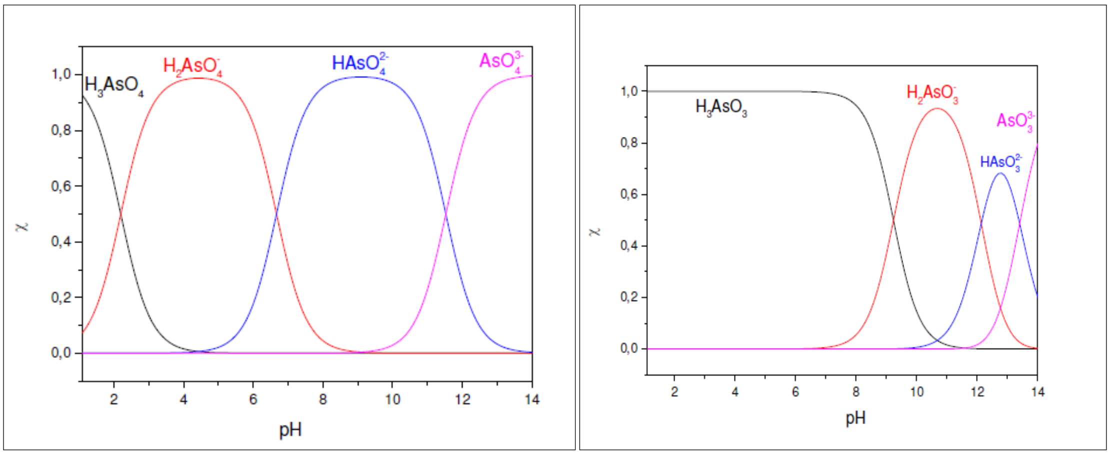

Figure S6. Bjerrum plot of arsenate (left) and arsenite (right) species reconstructed employing the dissociation constants of arsenic acid ($pKa_1 = 2.20$; $pKa_2 = 6.67$; $pKa_3 = 11.53$) and arsenious acid ($pKa_1 = 9.23$; $pKa_2 = 12.13$; $pKa_3 = 13.40$).

Sorbent surface in water

$$\equiv Fe - OH + H_2O \rightleftarrows \equiv Fe - OH_2^+ + OH^-$$

$$\equiv Fe - OH + H_2O \rightleftarrows \equiv Fe - O^- + H_3O^+$$

$$\equiv Fe - O^- + H_2O \rightleftarrows \equiv Fe - OH + OH^-$$

$$\equiv Fe - OH_2^+ + H_2O \rightleftarrows \equiv Fe - OH + H_3O^+$$

$$\equiv Fe^+ + 2H_2O \rightleftarrows \equiv Fe - OH + H_3O^+$$

$$\equiv Si - OH + H_2O \rightleftarrows \equiv Si - O^- + H_3O^+$$

Figure S7. Reactions of the sorbent surface in water.

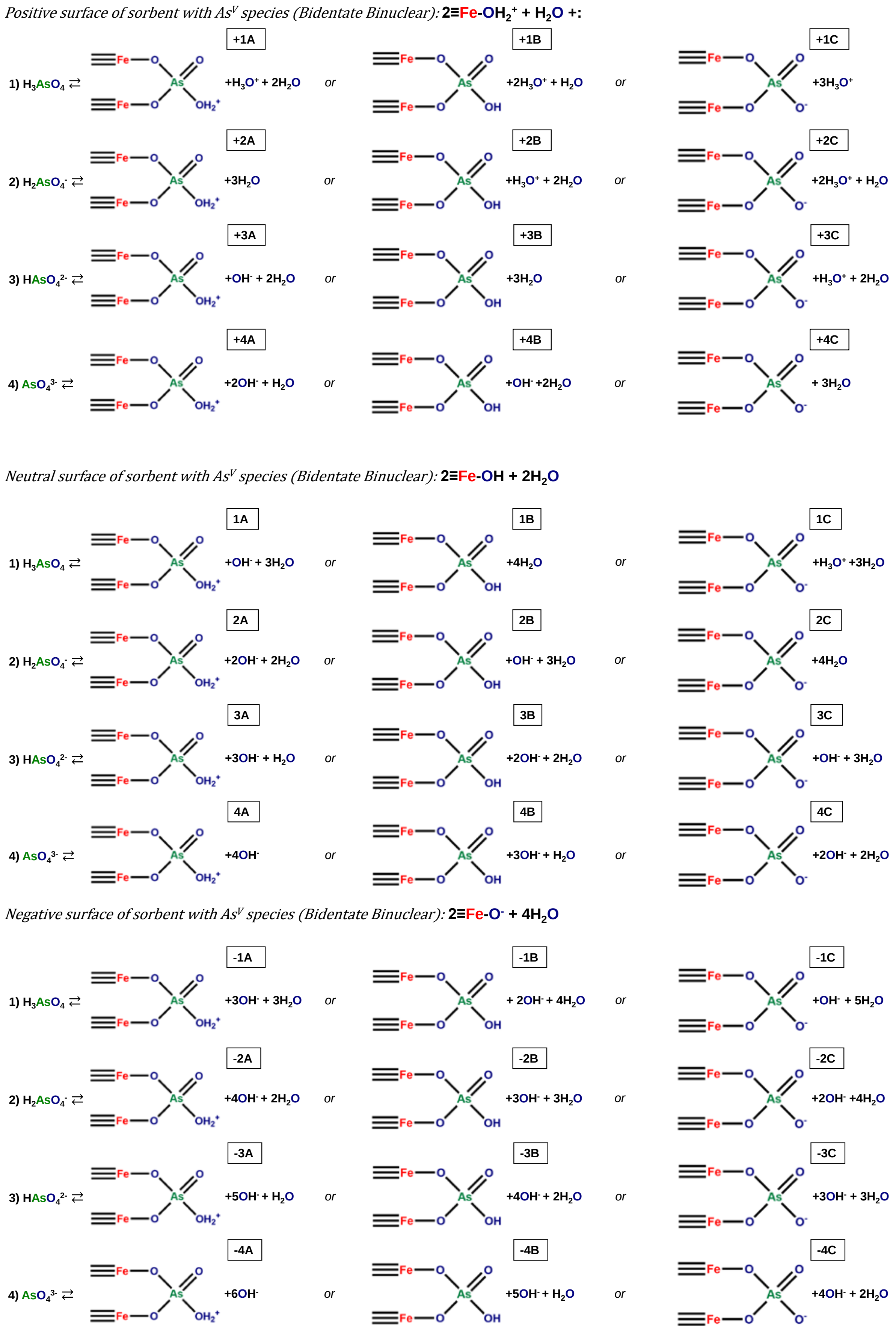

Figure S8. Possible reactions between the sorbent surface and arsenate species in water.

4. Adsorption tests: effect of pH As^V

Table S4. Batch experiments results of the sorbents at initial concentration of 100 or 500 mg L^{-1} of As^V at various pH. Volume of the contaminant was 20 mL and adsorption time was 16 hours.

Sorbent	Moisture (%)	m (mg)	V (mL)	pH_0	pH_{Int}	pH_{Fin}	C_0 (mg L^{-1})	C_e As^V (mg L^{-1})	q_e (mg/g)	As Ads (%)	q_e (mg/m²)	C_e Si (mg L^{-1})
Aka	7.24%	51.6	20	2.11	1.88	2.06	97.9	0.2	40.8	99.8%	0.202	*n.a.*
		49.2	20	2.93	2.65	2.61	95.5	0.2	41.8	99.8%	0.207	*n.a.*
		51.5	20	3.97	2.81	2.64	96.5	0.1	40.3	99.9%	0.200	*n.a.*
		51.2	20	6.14	3.07	2.81	96.7	0.1	40.7	99.9%	0.201	*n.a.*
		49.8	20	8.06	5.76	3.99	98.4	0.8	42.3	99.2%	0.209	*n.a.*
		49.8	20	2.15	1.90	1.81	459.0	258.3	86.9	43.7%	0.430	*n.a.*
		50.9	20	3.17	2.91	3.27	469.9	281.5	79.8	40.1%	0.395	*n.a.*
		49.8	20	3.05	2.89	2.82	484.2	279.7	88.5	42.2%	0.438	*n.a.*
		50.9	20	3.94	3.25	3.39	481.9	290.2	81.2	39.8%	0.402	*n.a.*
		50.9	20	6.16	6.28	6.41	481.2	339.3	60.1	29.5%	0.298	*n.a.*
		50.3	20	8.21	7.68	7.35	455.3	335.1	51.5	26.4%	0.255	*n.a.*
Fer	10.67%	45.5	20	2.09	2.15	2.05	104.7	0.4	45.9	99.7%	0.176	*n.a.*
		44.6	20	2.94	3.55	4.77	105.1	6.5	44.2	93.8%	0.170	*n.a.*
		45.3	20	3.98	6.46	7.03	105.8	46.2	26.3	56.3%	0.101	*n.a.*
		45.3	20	6.04	6.82	7.20	106.1	52.9	23.5	50.1%	0.090	*n.a.*
		45.3	20	8.02	8.28	8.40	105.0	80.9	10.6	22.9%	0.041	*n.a.*
		44.8	20	2.09	2.24	1.96	512.6	353.4	71.0	31.1%	0.273	*n.a.*
		45.0	20	3.05	3.32	4.33	518.5	379.4	61.9	26.8%	0.238	*n.a.*
		44.7	20	4.00	5.61	6.08	517.6	409.9	48.1	20.8%	0.185	*n.a.*
		45.3	20	5.96	6.15	6.28	520.7	414.7	46.8	20.4%	0.180	*n.a.*
		45.2	20	8.07	8.23	8.26	516.6	464.0	23.3	10.2%	0.089	*n.a.*
Mag	1.31%	49.9	20	2.22	2.18	2.20	92.3	29.1	25.7	68.5%	0.279	*n.a.*
		51.3	20	3.19	3.53	3.73	93.2	44.0	19.4	52.8%	0.211	*n.a.*
		51.5	20	3.98	6.23	5.81	93.7	60.9	12.9	35.0%	0.140	*n.a.*
		50.1	20	6.20	6.70	6.71	93.3	66.8	10.7	28.4%	0.117	*n.a.*
		50.1	20	8.11	8.35	8.07	94.6	79.3	6.2	16.2%	0.068	*n.a.*
Comp	2.28%	49.6	20	2.22	*n.a.*	2.26	98.4	79.8	7.7	18.9%	0.019	5.19
		50.5	20	3.20	*n.a.*	3.49	101.7	86.9	6.0	14.6%	0.015	7.36
		50.0	20	4.22	*n.a.*	6.30	103.9	92.0	4.9	11.4%	0.012	20.35
		50.6	20	6.15	*n.a.*	5.08	101.0	93.2	3.1	7.7%	0.008	32.19
		51.1	20	8.07	*n.a.*	7.10	101.3	95.2	2.5	6.1%	0.006	46.60

pH_0 is the pH of the solution before contact with the sorbent. pH_{int} is the pH of the solution immediately after contact with the sorbent. pH_{fin} is the pH of the solution after adsorption test and separation of the sorbent from the liquid. C_0 is the initial arsenic concentration. C_e is the final arsenic concentration. q_e is the adsorbed amount of arsenic, calculated with Eq.1. Relative errors (E_r): E_r(Moisture) = 2%; E_r(m) = 1%; E_r(V) = 1%; E_r(pH) = 2%; E_r(C) = 2%; E_r(q_e) = 7%.

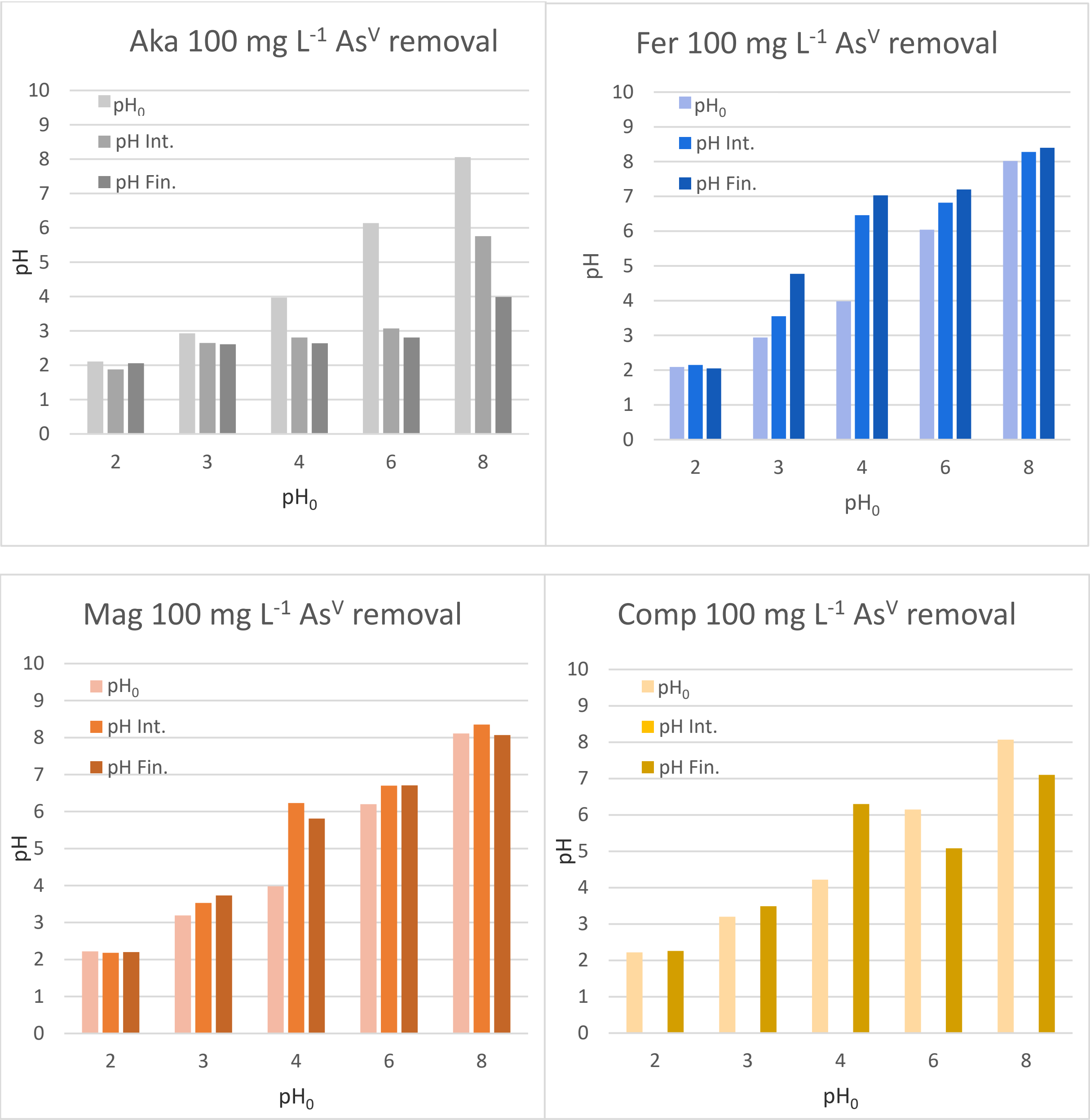

Figure S9. Evolution of initial pH (pH_0), intermediate pH (pH_{Int}) and final pH (pH_{Fin}) for various starting pH (pH_0) for the sorbents with initial concentration of As^V equal to 100 mg L^{-1}.

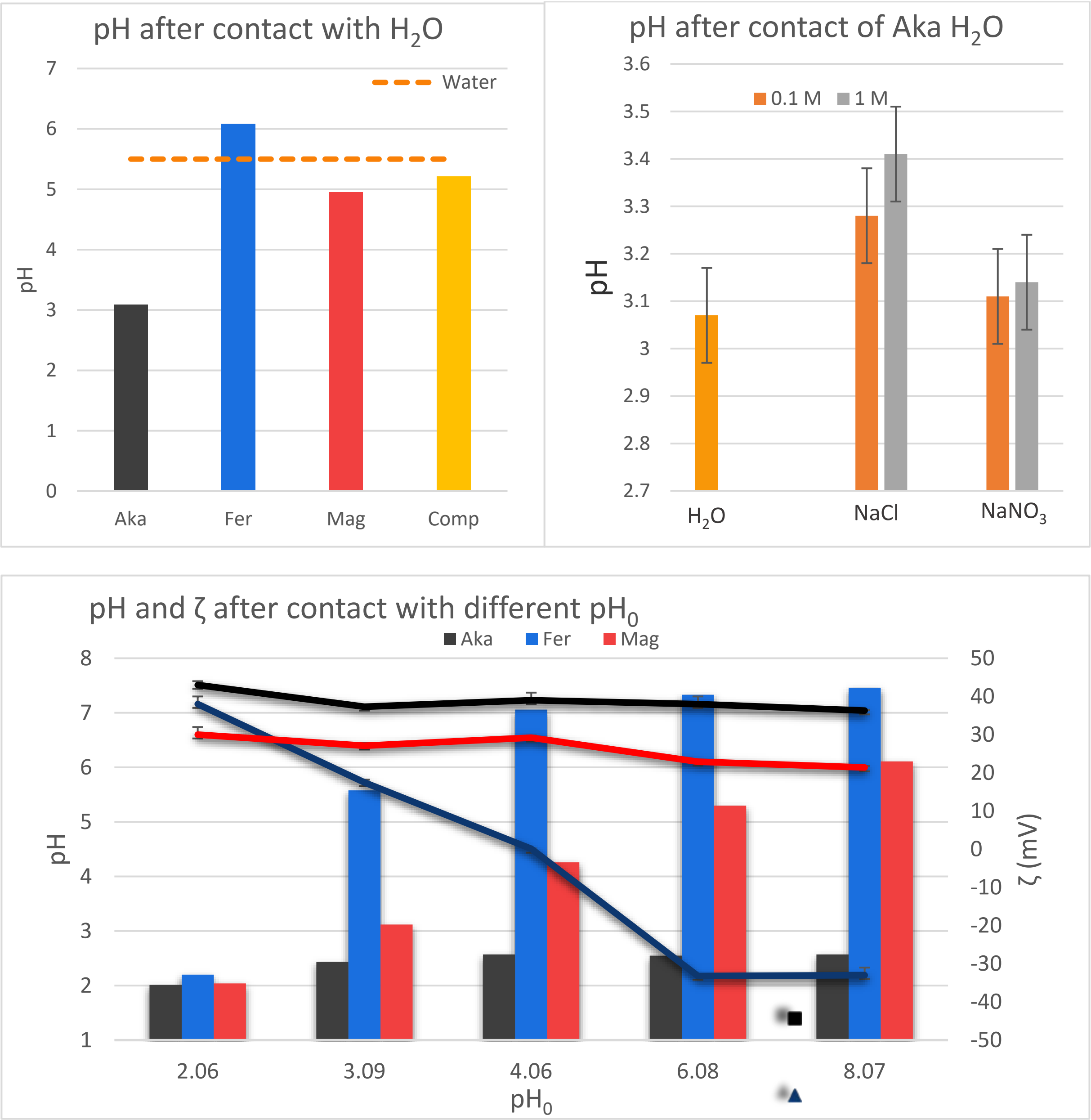

Figure S10. pH of solution after contact with sorbents.

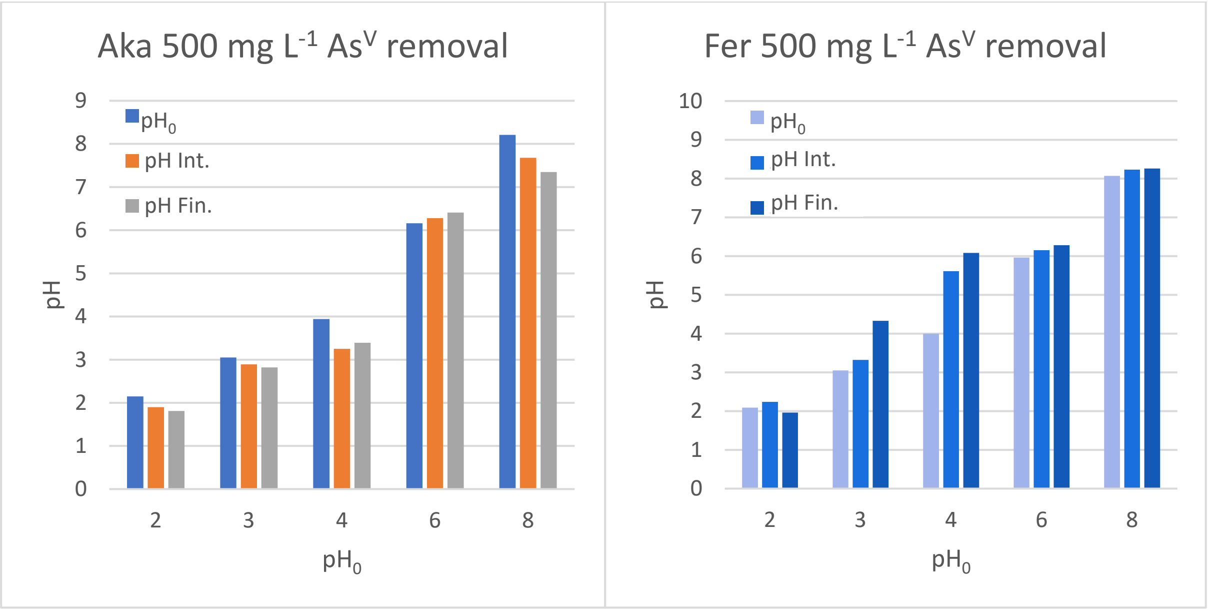

Figure S11. Evolution of initial pH (pH_0), intermediate pH (pH_{Int}) and final pH (pH_{Fin}) for various starting pH (pH_0) for the sorbents with initial concentration of As^V equal to 500 mg L^{-1}.

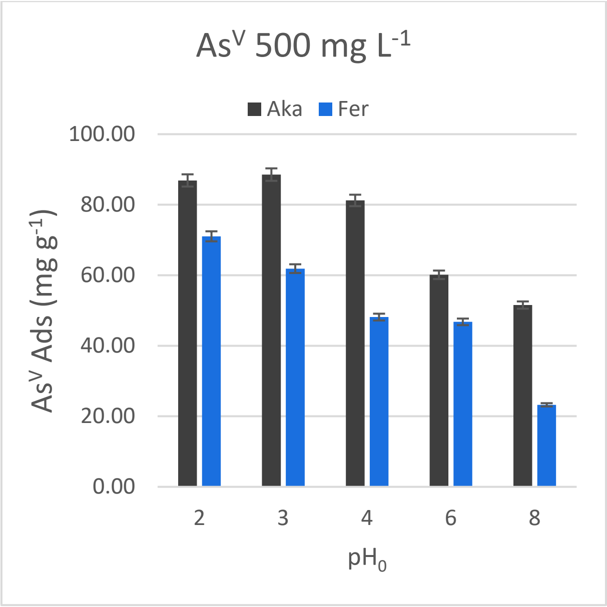

Figure S12. Adsorption capacity from batch adsorption experiments with 500 mg L^{-1} As^V solution on Aka (black) and Fer (blue) at different initial pH (pH_0).

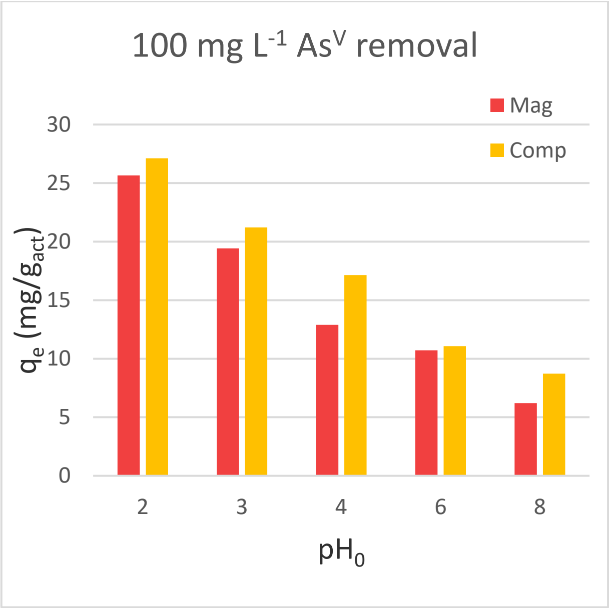

Figure S13. Adsorption capacity from batch adsorption experiments with 100 mg L^{-1} As^{V} solution on maghemite and composite normalized for its active phase (28.3%) at different initial pH (pH_0).

5. Adsorption tests: effect of pH As^{III}

Table S5. Batch experiments results of the sorbents at initial concentration of 100 or 500 mg L^{-1} of As^{III} at various pH. Volume of the contaminant was 20 mL and adsorption time was 16 hours.

Sorbent	Moisture (%)	m (mg)	V (mL)	pH_0	pH_{Int}	pH_{Fin}	C_0 (mg L^{-1})	C_e As^{III} (mg L^{-1})	q_e (mg/g)	As Ads (%)	q_e (mg/m²)	C_e Si (mg L^{-1})
**Aka**	**7.24%**	49.8	20	2.12	1.78	1.72	102.6	37.2	28.3	63.8%	0.140	*n.a.*
		50.8	20	3.06	2.72	2.61	103.9	19.5	35.8	81.2%	0.177	*n.a.*
		49.3	20	4.13	2.86	2.68	103.9	18.4	37.4	82.3%	0.185	*n.a.*
		49.4	20	6.05	2.91	2.89	103.1	20.8	35.9	79.8%	0.178	*n.a.*
		50.8	20	8.04	2.95	2.90	102.9	17.0	36.4	83.4%	0.180	*n.a.*
		46.6	20	2.04	2.06	2.07	518.3	364.9	65.9	29.6%	0.326	*n.a.*
		46.6	20	2.97	2.99	2.49	567.1	355.8	90.7	37.3%	0.449	*n.a.*
		46.8	20	4.09	3.14	2.63	549.5	351.1	84.8	36.1%	0.420	*n.a.*
		46.8	20	6.05	3.06	2.43	564.6	352.9	90.5	37.5%	0.448	*n.a.*
		46.7	20	7.98	3.33	2.56	570.6	356.9	91.4	37.5%	0.453	*n.a.*
**Fer**	**10.67%**	45.0	20	2.10	2.14	2.20	115.8	14.5	45.1	87.5%	0.173	*n.a.*
		45.2	20	2.99	3.25	3.17	119.8	7.1	49.9	94.1%	0.192	*n.a.*
		44.7	20	4.08	5.03	5.05	119.7	5.1	51.2	95.7%	0.197	*n.a.*
		44.8	20	6.01	6.12	5.91	119.3	4.5	51.3	96.2%	0.197	*n.a.*
		45.5	20	8.05	6.83	6.65	119.2	4.3	50.6	96.4%	0.194	*n.a.*
		50.37	20	2.07	2.16	1.89	544.2	293.0	111.7	46.2%	0.429	*n.a.*
		50.19	20	2.97	3.47	3.08	567.1	260.6	136.7	54.0%	0.526	*n.a.*
		50.33	20	4.09	5.20	4.78	549.5	248.6	133.9	54.8%	0.515	*n.a.*
		50.30	20	6.05	6.16	5.78	564.6	248.4	140.7	56.0%	0.541	*n.a.*
		50.37	20	7.98	7.45	6.77	570.6	245.8	144.4	56.9%	0.555	*n.a.*
**Mag**	**1.31%**	52.0	20	2.14	2.15	1.92	99.5	57.8	16.3	41.9%	0.177	*n.a.*
		51.5	20	3.15	3.09	2.90	98.7	48.9	19.6	50.4%	0.213	*n.a.*
		50.0	20	3.99	4.17	5.09	98.1	45.1	21.5	54.1%	0.234	*n.a.*
		50.3	20	6.16	5.88	7.11	97.8	44.2	21.6	54.9%	0.235	*n.a.*
		50.8	20	8.02	6.77	7.68	98.6	41.2	22.9	58.2%	0.249	*n.a.*
**Comp**	**2.28%**	50.8	20	2.18	1.97	2.20	97.7	93.3	1.8	4.5%	0.004	4.39
		51.0	20	3.24	3.30	3.51	101.3	95.0	2.5	6.1%	0.006	5.56
		49.7	20	3.97	4.00	4.99	106.1	93.3	5.3	12.1%	0.013	7.42
		50.6	20	5.89	5.76	6.92	99.7	91.2	3.4	8.5%	0.008	12.78
		49.7	20	8.01	7.25	7.56	98.3	90.1	3.4	8.3%	0.008	28.59

pH_0 is the pH of the solution before contact with the sorbent. pH_{int} is the pH of the solution immediately after contact with the sorbent. pH_{fin} is the pH of the solution after adsorption test and separation of the sorbent from the liquid. C_0 is the initial arsenic concentration. C_e is the final arsenic concentration. q_e is the adsorbed amount of arsenic, calculated with Eq.1. Relative errors (E_r): E_r(Moisture) = 2%; E_r(m) = 1%; E_r(V) = 1%; E_r(pH) = 2%; E_r(C) = 2%; E_r(q_e) = 7%.

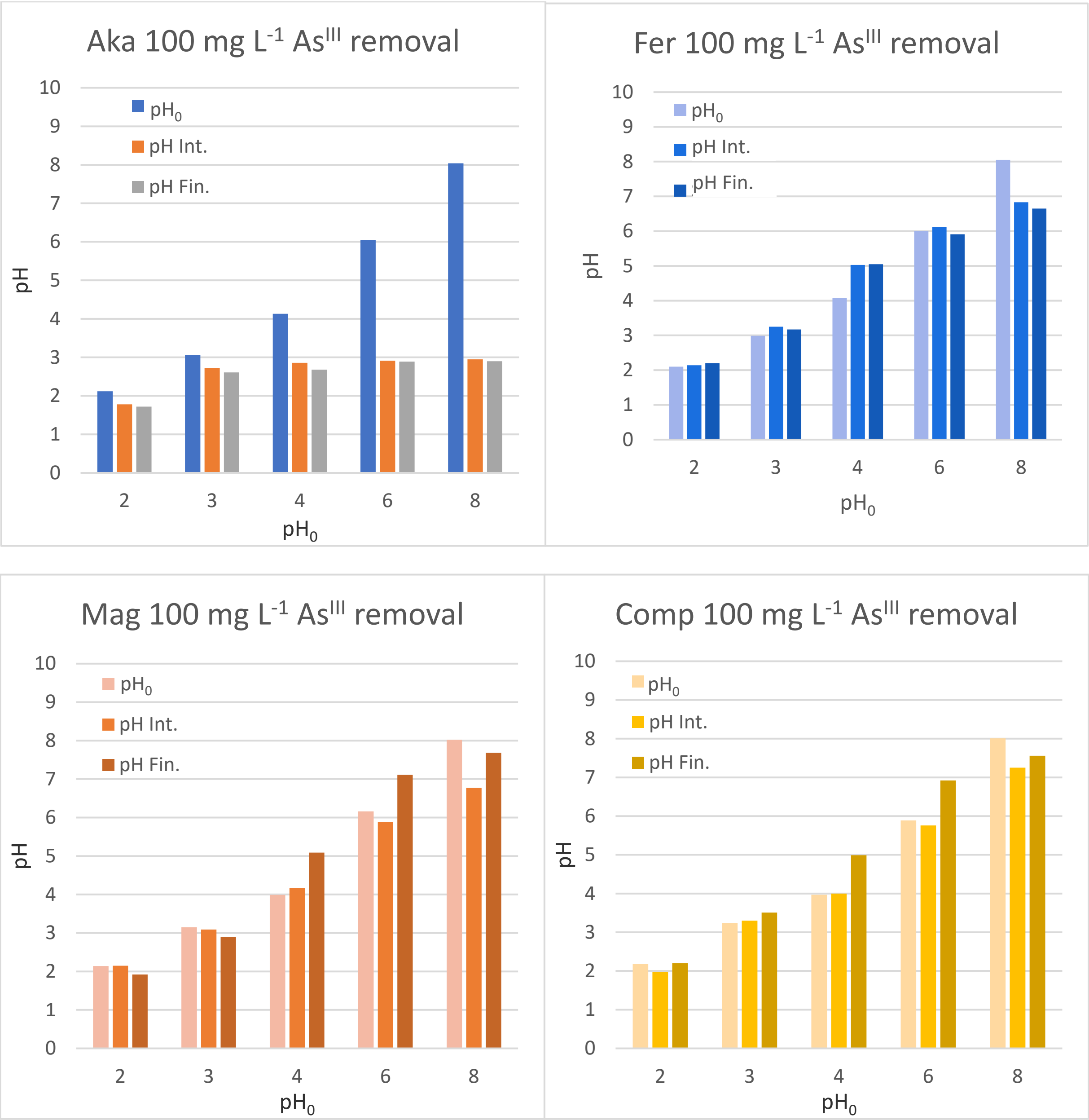

Figure S14. Evolution of initial pH (pH_0), intermediate pH (pH_{Int}) and final pH (pH_{Fin}) for various starting pH (pH_0) for the sorbents with initial concentration of As^{III} equal to 100 mg L^{-1}.

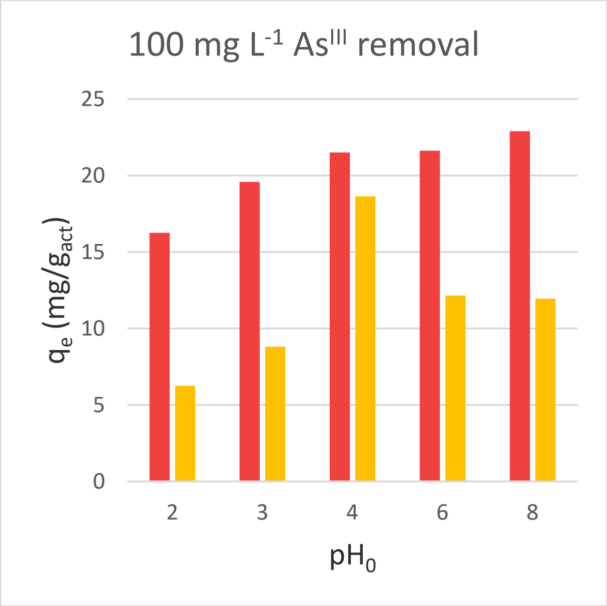

Figure S15. Adsorption capacity from batch adsorption experiments with 100 mg L^{-1} As^{III} solution on maghemite and composite normalized for its active phase (28.3%) at different initial pH (pH_0).

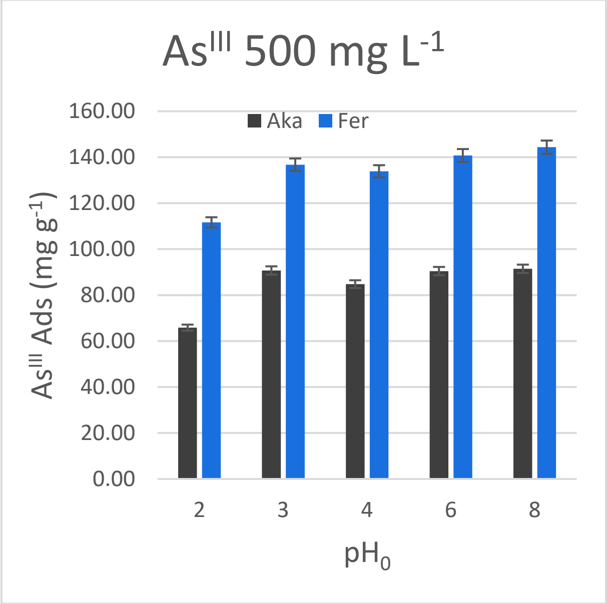

Figure S16. Adsorption capacity from batch adsorption experiments with 500 mg L^{-1} As^{III} solution on Aka (black) and Fer (blue) at different initial pH (pH_0).

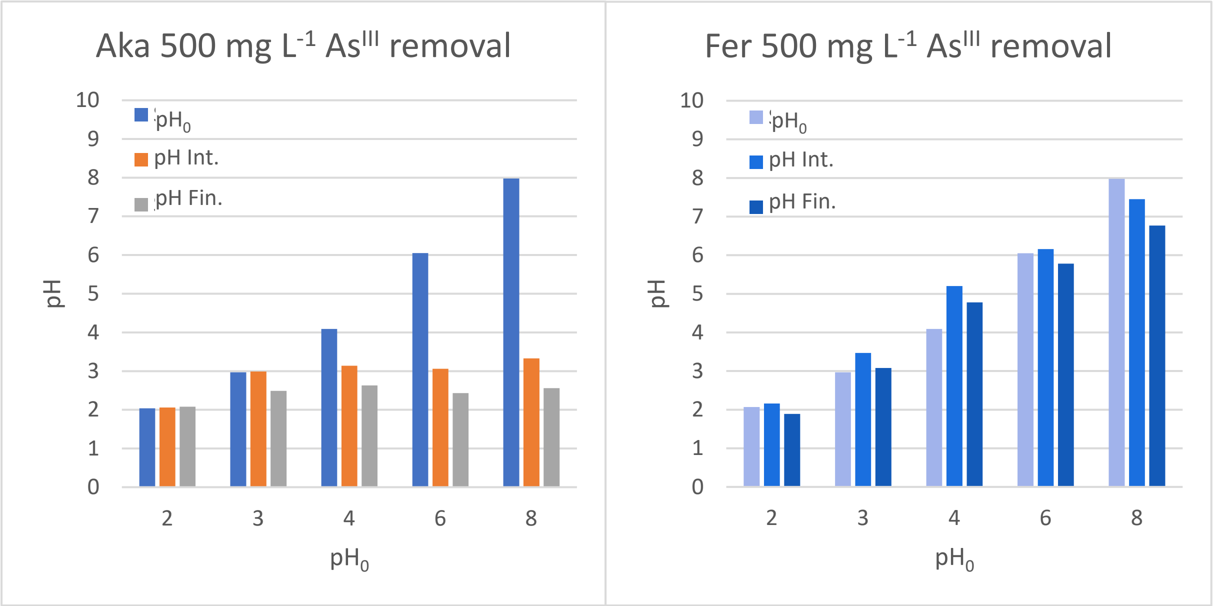

Figure S17. Evolution of initial pH (pH_0), intermediate pH (pH_{Int}) and final pH (pH_{Fin}) for various starting pH (pH_0) for the sorbents with initial concentration of As^{III} equal to 500 mg L^{-1}.

6. FTIR Spectra

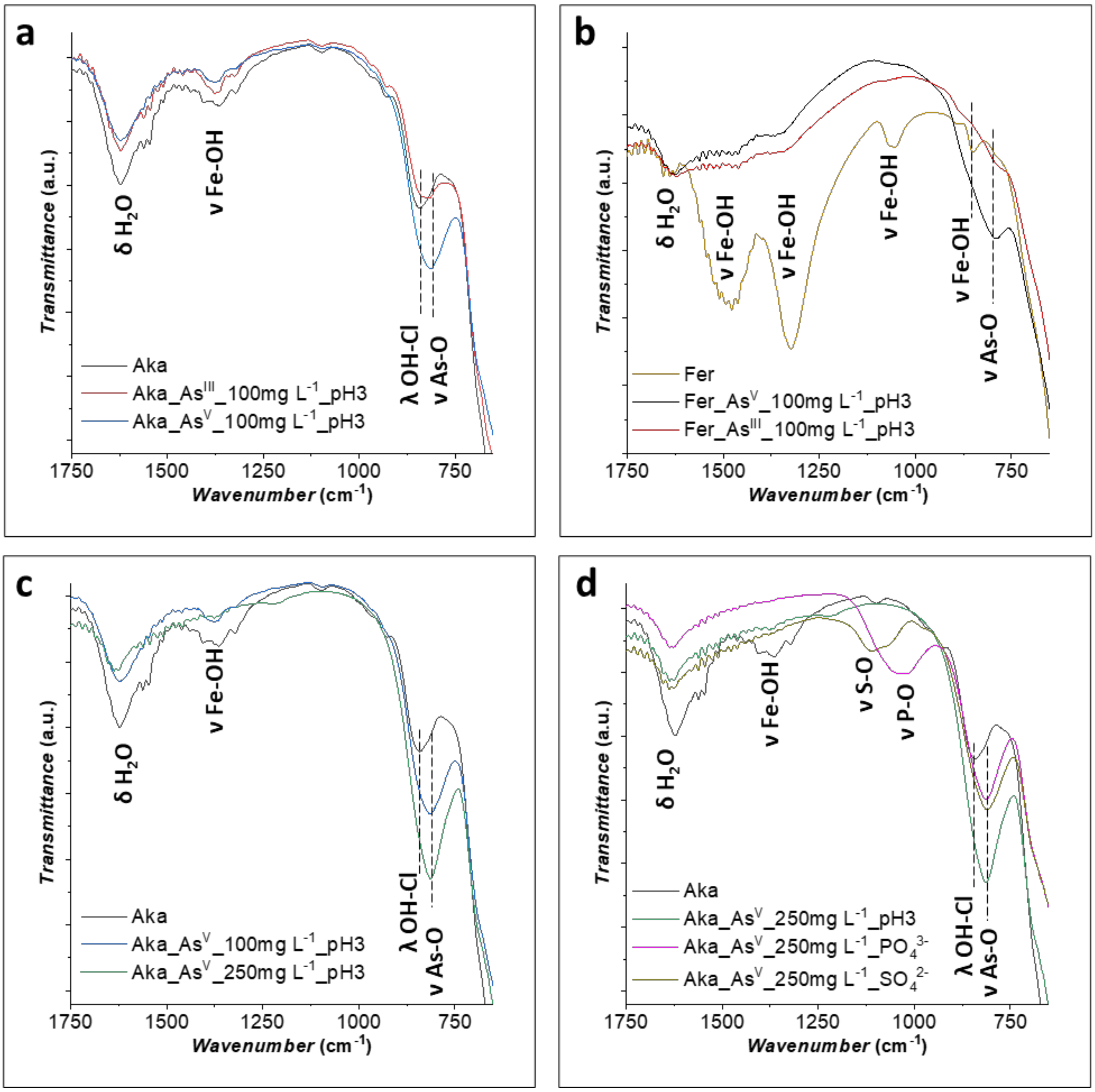

Figure S18. FTIR spectra of akaganeite and ferrihydrite after arsenic removal.

7. Adsorption tests: effect of initial concentration

Table S6. Batch experiments results of the sorbents at various initial concentration of of As^V at pH 3 and 8. Volume of the contaminant was 20 mL and adsorption time was 16 hours.

Sorbent	Moisture (%)	m (mg)	V (mL)	pH_0	pH_{Int}	pH_{Fin}	C_0 (mg L^{-1})	C_e As^V (mg L^{-1})	q_e (mg g^{-1})	As Ads (%)	q_e (mg m^{-2})
Aka	7.24%	49.9	20	2.99	2.52	2.59	10.0	0.0	4.3	99.7%	0.021
		50	20	3.13	2.82	2.65	46.8	0.0	20.2	99.9%	0.100
		49.2	20	2.93	2.65	2.61	95.5	0.2	41.8	99.8%	0.207
		49.5	20	3.12	2.69	2.68	145.3	3.9	61.6	97.3%	0.305
		51.5	20	3.01	2.77	2.67	205.9	20.8	77.5	89.9%	0.384
		50.3	20	2.95	2.63	2.84	255.6	74.7	77.5	70.8%	0.384
		51.4	20	3.03	2.81	2.79	363.0	151.4	88.8	58.3%	0.439
		49.8	20	3.05	2.89	2.82	484.2	279.7	88.5	42.2%	0.438
		51.1	20	7.94	2.76	2.63	9.9	0.1	4.2	99.4%	0.021
		51.1	20	8.03	3.09	2.84	45.7	0.1	19.3	99.9%	0.095
		49.8	20	8.06	5.76	3.99	98.4	0.8	42.3	99.2%	0.209
		51.2	20	8.14	6.53	5.53	142.4	31.3	46.8	78.0%	0.232
		49.9	20	8.01	6.72	6.38	212.8	104.5	46.8	50.9%	0.232
		49.9	20	8.17	7.32	6.91	242.1	130.3	48.3	46.2%	0.239
		50.4	20	8.04	7.12	6.97	376.8	264.8	47.9	29.7%	0.237
		50.3	20	8.21	7.68	7.35	455.3	335.1	51.5	26.4%	0.255

pH_0 is the pH of the solution before contact with the sorbent. pH_{int} is the pH of the solution immediately after contact with the sorbent. pH_{fin} is the pH of the solution after adsorption test and separation of the sorbent from the liquid. C_0 is the initial arsenic concentration. C_e is the final arsenic concentration. q_e is the adsorbed amount of arsenic, calculated with Eq.1. Relative errors (E_r): E_r(Moisture) = 2%; E_r(m) = 1%; E_r(V) = 1%; E_r(pH) = 2%; E_r(C) = 2%; $E_r(q_e)$ = 7%.

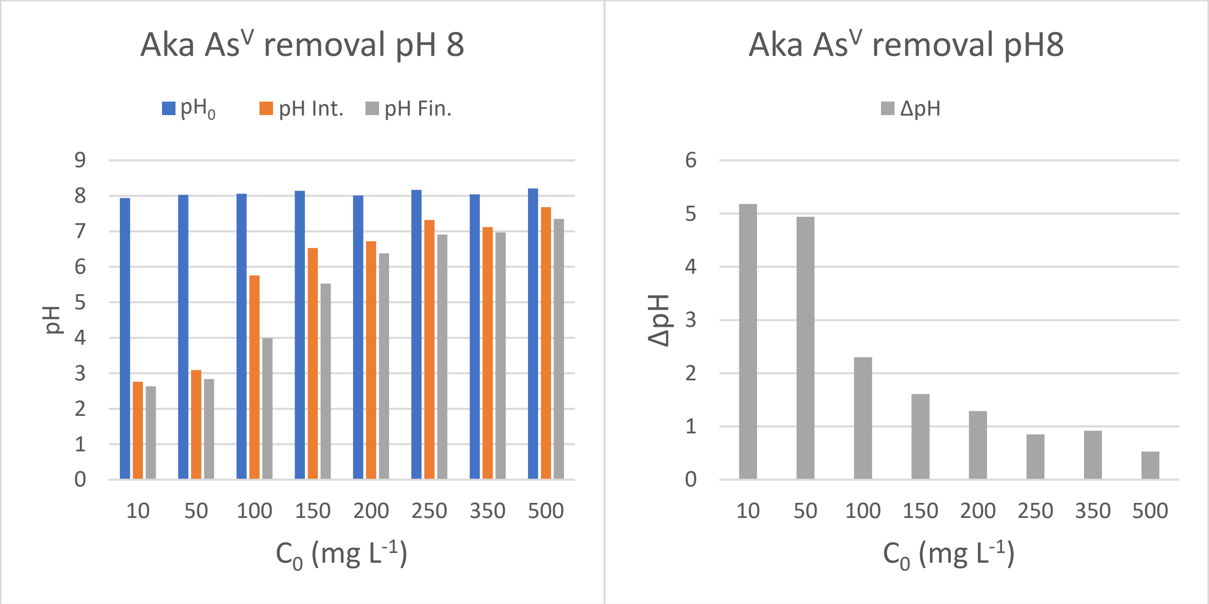

Figure S19. Evolution of initial pH (pH_0), intermediate pH (pH_{Int}) and final pH (pH_{Fin}) (left) and ΔpH between intermediate and initial pH (right) at various As^V initial concentration for Aka.

8. Adsorption tests: effect of contact time

Table S7. Batch experiments results of the sorbents at various contact time with As^V at pH 3 and 8. Volume of the contaminant was 20 mL initial concentration of about 250 mg L^{-1}.

Sorbent	Moisture (%)	m (mg)	V (mL)	t (min)	pH_0	$pH_{Fin.}$	C_0 (mg L^{-1})	C_e As^V (mg L^{-1})	q_e (mg g^{-1})	As Ads (%)	q_e (mg m^{-2})
Aka	7.24%	50.0	20	10	3.14	3.15	269.8	126.6	61.7	53.1%	0.306
		50.2	20	30	3.14	3.26	269.8	111.5	68.0	58.7%	0.337
		49.5	20	60	3.14	3.22	269.8	112.6	68.5	58.3%	0.339
		50.1	20	120	3.14	2.78	269.8	100.9	72.7	62.6%	0.360
		50.6	20	240	3.14	2.90	269.8	96.1	74.0	64.4%	0.367
		50.3	20	960	3.14	2.81	255.6	74.7	77.5	70.8%	0.384
		50.2	20	10	7.97	6.79	263.6	173.7	38.6	34.1%	0.191
		50.7	20	30	7.97	6.72	263.6	164.0	42.3	37.8%	0.210
		50.3	20	60	7.97	6.81	263.6	158.7	45.0	39.8%	0.223
		50.7	20	120	7.97	6.60	263.6	166.6	41.2	36.8%	0.204
		49.6	20	240	7.97	6.50	263.6	148.5	50.0	43.7%	0.248
		49.9	20	960	8.17	6.91	242.1	130.3	48.3	46.2%	0.239

pH_0 is the pH of the solution before contact with the sorbent. pH_{fin} is the pH of the solution after adsorption test and separation of the sorbent from the liquid. C_0 is the initial arsenic concentration. C_e is the final arsenic concentration. q_t is the adsorbed amount of arsenic at a certain time, calculated with Eq.8. Relative errors (E_r): E_r(Moisture) = 2%; E_r(m) = 1%; E_r(V) = 1%; E_r(pH) = 2%; E_r(C) = 2%; E_r(q_e) = 7%.

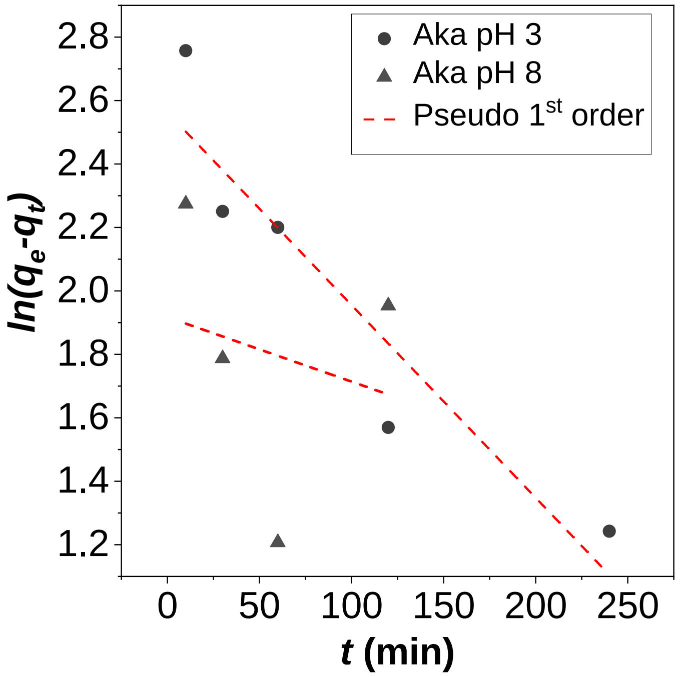

Figure S20. Sorption kinetics of As^V on Aka at pH 3 and 8 fitted by linearized pseudo 1st order fitting.

Table S8. Linear pseudo 2nd order and intraparticle diffusion models fitting parameters for adsorption of As^V onto Aka at pH_0 3 and 8.

Sample	pH_0	q_e^{exp} (mg g^{-1})	$q_e''^{calc}$ (mg g^{-1})	K'' (g mg^{-1} min^{-1})	R^2	k_i (g mg^{-1} min$^{-0.5}$)	x_i (mg g^{-1})	R^2
**Aka**	3	78(5)	78.0(4)	$1.8(5)\cdot10^{-3}$	0.99	1.5(7); 0.24(1)	58(4); 70.2(3)	0.81; 0.99
	8	48(3)	48.7(6)	$4(3)\cdot10^{-3}$	0.99	1.4(1); 0.2(4)	34.3(8); 42(8)	0.99; 0.28

9. Adsorption tests: effect of ionic strength

Table S9. Batch experiments results of the sorbents at different ionic strengh with As^V at pH 3 and 8. Volume of the contaminant was 20 mL initial concentration of about 250 mg L^{-1}. pH_0 is the pH of the solution before contact with the sorbent.

Sorbent	Moisture (%)	m (mg)	V (mL)	NaCl (M)	pH_0	pH_{Int}	pH_{Fin}	C_0 As^V (mg L^{-1})	C_e As^V (mg L^{-1})	q_e (mg g^{-1})	As Ads (%)	q_e (mg m^{-2})
Aka	7.24%	50.3	20	0	3.14	2.83	2.81	255.6	74.7	77.5	70.8%	0.384
		50.7	20	0.01	3.01	2.8	3.06	262.9	89.0	74.0	66.2%	0.366
		50.3	20	0.1	3.12	2.89	2.95	256.7	86.0	73.2	66.5%	0.362
		50.5	20	1	3.16	2.98	2.86	284.6	120.1	70.3	57.8%	0.348
		49.9	20	0	8.17	7.32	6.91	242.1	130.3	48.3	46.2%	0.239
		50.7	20	0.01	8.12	6.88	6.57	260.4	138.6	51.8	46.8%	0.257
		49.7	20	0.1	7.98	6.98	7.02	252.7	126.3	54.8	50.0%	0.272
		50.7	20	1	8.08	7.09	6.64	299.7	153.3	62.3	48.9%	0.308

pH_{fin} is the pH of the solution after adsorption test and separation of the sorbent from the liquid. C_0 is the initial arsenic concentration. C_e is the final arsenic concentration. q_e is the adsorbed amount of arsenic at a certain time, calculated with Eq.1. Relative errors (E_r): E_r(Moisture) = 2%; E_r(m) = 1%; E_r(V) = 1%; E_r(pH) = 2%; E_r(C) = 2%; E_r(q_e) = 7%.

10. Adsorption tests: effect of competitors

Table S10. Batch experiments results of the sorbents at different sulfate competitor concentration with As^V at pH 3 and 8. Volume of the contaminant was 20 mL initial concentration of about 250 mg L^{-1}.

Sorbent	Moisture (%)	M (mg)	V (mL)	SO_4^{2-} (M)	pH_0	pH_{Int}	pH_{Fin}	C_0 (mg L^{-1})	C_e As^V (mg L^{-1})	q_e (mg g^{-1})	As Ads (%)	q_e (mg m^{-2})
Aka	7.24%	50.3	20	0.00	3.14	2.83	2.81	255.6	74.7	77.5	70.8%	0.384
		49.6	20	0.003	3.11	3.2	2.92	259.2	105.2	66.9	59.4%	0.331
		50.6	20	0.033	3.04	3.12	2.62	274.9	125.7	63.6	54.3%	0.315
		50.7	20	0.334	3.15	3.18	2.76	252.8	137.6	49.0	45.6%	0.243
		49.9	20	0.00	8.17	7.32	6.91	242.1	130.3	48.3	46.2%	0.239
		50.8	20	0.003	8.07	7.09	6.49	260.9	131.1	55.1	49.8%	0.273
		49.6	20	0.033	7.94	7.1	6.55	267.8	131.1	59.4	51.1%	0.294
		50.2	20	0.334	7.97	7.38	6.51	273.8	142.8	56.2	47.8%	0.278

pH_0 is the pH of the solution before contact with the sorbent. pH_{fin} is the pH of the solution after adsorption test and separation of the sorbent from the liquid. C_0 is the initial arsenic concentration. C_e is the final arsenic concentration. q_e is the adsorbed amount of arsenic at a certain time, calculated with Eq.1. Relative errors (E_r): E_r(Moisture) = 2%; E_r(m) = 1%; E_r(V) = 1%; E_r(pH) = 2%; E_r(C) = 2%; $E_r(q_e)$ = 7%.

Table S11. Batch experiments results of the sorbents at different phosfate concentration with As^V at pH 3 and 8. Volume of the contaminant was 20 mL initial concentration of about 250 mg L^{-1}.

Sorbent	Moisture (%)	m (mg)	V (mL)	PO_4^{3-} (M)	pH_0	pH_{Int}	pH_{Fin}	C_0 (mg L^{-1})	C_e As^V (mg L^{-1})	q_e (mg g^{-1})	As Ads (%)	q_e (mg m^{-2})
Aka	7.24%	50.3	20	0.00	3.14	2.83	2.81	255.6	74.7	77.5	70.8%	0.384
		49.3	20	0.003	3.16	2.83	2.99	264.2	125.4	60.7	52.5%	0.301
		50.4	20	0.033	3.16	3.01	3.36	251.8	175.0	32.9	30.5%	0.163
		50.1	20	0.334	3.15	3.27	3.43	166.6	146.1	8.8	12.3%	0.044
		49.9	20	0.00	8.17	7.32	6.91	242.1	130.3	48.3	46.2%	0.239
		49.6	20	0.003	7.96	7.27	7.15	267.6	169.8	42.5	36.5%	0.210
		50.5	20	0.033	8.09	7.91	7.98	262.4	207.6	23.4	20.9%	0.116
		50.1	20	0.334	8.06	8.11	8.18	201.1	203.3	0.0	0.0%	0.000

pH_0 is the pH of the solution before contact with the sorbent. pH_{fin} is the pH of the solution after adsorption test and separation of the sorbent from the liquid. C_0 is the initial arsenic concentration. C_e is the final arsenic concentration. q_e is the adsorbed amount of arsenic at a certain time, calculated with Eq.1. Relative errors (E_r): E_r(Moisture) = 2%; E_r(m) = 1%; E_r(V) = 1%; E_r(pH) = 2%; E_r(C) = 2%; $E_r(q_e)$ = 7%.
